# Switchable encapsulation of polysulfides in the transition between sulfur and lithium sulfide

**DOI:** 10.1038/s41467-020-14686-2

**Published:** 2020-02-12

**Authors:** Yongsheng Fu, Zhen Wu, Yifei Yuan, Peng Chen, Lei Yu, Lei Yuan, Qiurui Han, Yingjie Lan, Wuxin Bai, Erjun Kan, Chengxi Huang, Xiaoping Ouyang, Xin Wang, Junwu Zhu, Jun Lu

**Affiliations:** 1 Key Laboratory for Soft Chemistry and Functional Materials of Ministry of Education, Nanjing University of Science and Technology, Nanjing, 210094 China; 2 Chemical Sciences and Engineering Division, Argonne National Laboratory, 9700 S. Cass Avenue, Lemont, IL 60439 USA; 3 Key Laboratory of Low Dimensional Materials and Application Technology, School of Materials Science and Engineering, Xiangtan University, Xiangtan, 411105 China

**Keywords:** Batteries, Batteries

## Abstract

Encapsulation strategies are widely used for alleviating dissolution and diffusion of polysulfides, but they experience nonrecoverable structural failure arising from the repetitive severe volume change during lithium−sulfur battery cycling. Here we report a methodology to construct an electrochemically recoverable protective layer of polysulfides using an electrolyte additive. The additive nitrogen-doped carbon dots maintain their “dissolved” status in the electrolyte at the full charge state, and some of them function as active sites for lithium sulfide growth at the full discharge state. When polysulfides are present amid the transition between sulfur and lithium sulfide, nitrogen-doped carbon dots become highly reactive with polysulfides to form a solid and recoverable polysulfide-encapsulating layer. This design skilfully avoids structural failure and efficiently suppresses polysulfide shuttling. The sulfur cathode delivers a high reversible capacity of 891 mAh g^−1^ at 0.5 C with 99.5% coulombic efficiency and cycling stability up to 1000 cycles at 2 C.

## Abstract (web summary)

Inspired by the processes of thrombus formation and thrombolysis in blood vessels, the authors here construct an electrochemically recoverable protective layer of polysulfides using an electrolyte additive, realizing high performance Li–S batteries.

## Introduction

With the rapid development of electronic devices and electric vehicles, the demand for high-energy-density batteries has increased significantly over the past few decades^1–3^. The lithium−sulfur (Li–S) battery is the most promising candidate to replace traditional lithium-ion batteries due to its high theoretical cathode capacity (1675 mAh g^−1^) and energy density (2500 Wh kg^−1^)^4,5^. Although significant improvements have been made in recent decades, serious challenges remain. The most notorious challenge is the rapid capacity decay and low coulombic efficiency that results when lithium polysulfides (LiPS) dissolve and diffuse in the electrolyte during the discharge/charge processes^6^.

Research on alleviating LiPS dissolution has focused on encapsulation in the sulfur electrodes of Li–S batteries. Carbon-based materials such as micro/mesoporous carbons, carbon nanotubes, carbon nanofibres, hollow carbon spheres, and graphene are widely used to confine LiPS by physical encapsulation^7–15^. To further enhance the interaction with polar LiPS, polar materials such as functionalized carbon materials (amino-modified reduced graphene oxide, nitrogen-doped mesoporous carbon), metal oxides, metal carbides, metal sulfides, metal nitrides, and polyoxometalates have also been investigated due to their strong chemical adsorption capacities^16–27^. These encapsulation strategies are generally effective at alleviating LiPS dissolution. However, because sulfur particles are preloaded to tightly fit the host geometry, they experience nonrecoverable structural failure arising from repetitive severe volume change during S-Li_2_S cycling. It is reasonably hypothesized that electrochemically constructing a recoverable protective layer at the electrode–electrolyte interface during S-Li_2_S cycling only when LiPS are present will solve this problem.

**Fig. 1:**
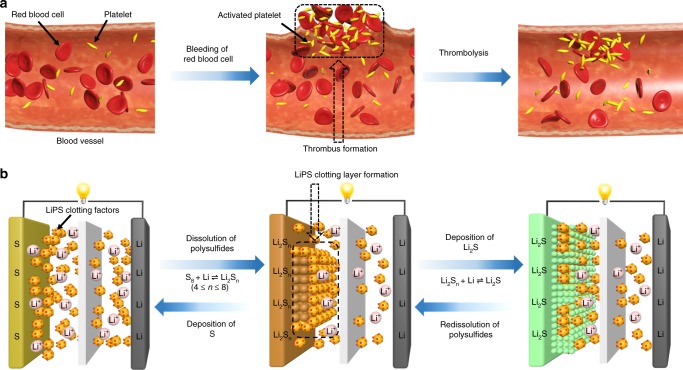
Schematics of the blood coagulation and LiPS clotting mechanisms. **a** In mammals, when an injury happens in a vessel wall, the platelets become activated, triggering the formation of thrombus to stanch bleeding. After the wound is healed, thrombolysis occurs spontaneously. **b** Diffusion of LiPS in Li–S batteries can be effectively restrained by a recoverable protective clotting layer formed in situ from the reaction between dissolved LiPS and LiPS clotting factors at the electrode–electrolyte interface during the discharge/charge process.

Technology is often inspired by nature. It is well known that hemostasis is a host’s defence mechanism to preserve the integrity of the closed high-pressure circulatory system when bleeding occurs. This complex process involves several steps: vasoconstriction, platelet activation, thrombus formation, and thrombolysis. Among them, thrombus formation and thrombolysis are self-healing and recoverable processes in the blood vessel^28–30^. As shown in Fig. 1a, when an injury occurs, the platelets in the blood are activated and start to form a platelet plug or thrombus, which can sufficiently seal the damaged blood vessel wall to restrict bleeding. When the injured blood vessel is completely healed, the signal of dissolution of the clot is automatically received in the blood vessel, and the body recovers homeostasis.

The recoverable processes of thrombus formation and thrombolysis in the blood vessel inspired us to explore a unique LiPS encapsulation strategy where a skilfully designed electrolyte additive (analogous to the platelet) can interact only with LiPS to form a recoverable protective layer in situ. Benefiting from the additive’s existence within the electrolyte, the protective layer is skilfully formed at the electrode–electrolyte interface rather than tightly surrounding each single sulfur particle, as previously reported in most studies. In addition, since the additive otherwise maintains its dissolved state when the electrode presents sulfur, the in situ formed protective layer thus senses no structural damage caused by the volume change of the sulfur electrode during S-Li_2_S transitions. The LiPS “clotting factors” (LiPSCFs) additive should meet at least three criteria: good dispersibility in the electrolyte, strong interaction with LiPS, and excellent restraining ability of the recoverable protective layer for the subsequent dissolution of LiPS.

Here in the present study, nitrogen-doped carbon dots (N-CDs) are chosen as LiPSCFs due to their low cost, facile fabrication and excellent dispersibility in electrolyte. The recoverable protective clotting layer of LiPS/N-CDs is clearly visualized in Li–S batteries both ex situ and in situ and blocks the subsequent dissolution and diffusion of LiPS. More interestingly, both discharge and charge involve the formation and dissolution of a protective LiPS/N-CDs clotting layer, similar to the recoverable processes of thrombus formation and thrombolysis in blood vessels but with N-CDs functioning as activated platelets. As a result, this design efficiently avoids structural failure caused by the large volume change of the sulfur electrode and suppresses LiPS shuttling. Battery testing shows that the sulfur cathode can deliver a high reversible capacity of 891 mAh g^−1^ at 0.5 C with 99.5% coulombic efficiency and cycling stability up to 1000 cycles.

## Results

### The strong interaction between the N-CDs and electrolyte and the formation of the LiPS/N-CDs clotting layer

**Fig. 2:**
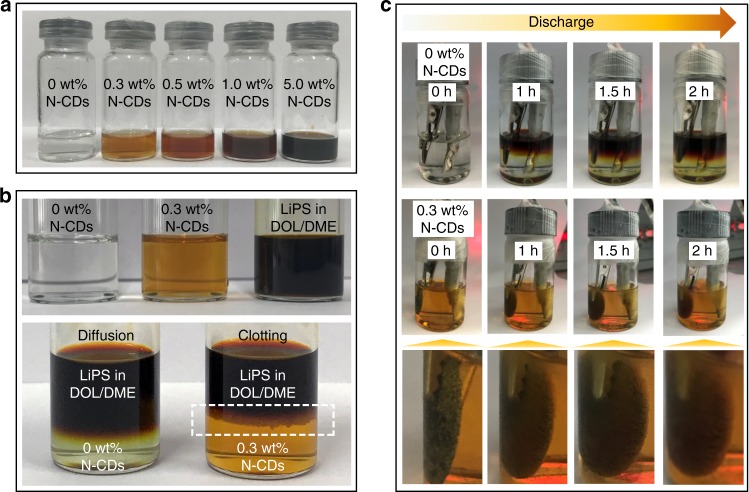
Photographs showing the formation of chemical and electrochemical LiPS/N-CDs clotting layers. **a** Electrolyte with different N-CD contents (0, 0.3, 0.5, 1.0, and 5.0 wt%). **b** Interaction between LiPS and electrolyte containing 0 and 0.3 wt% N-CDs. The white dashed frame highlights the formation of the protective layer at the LiPS/electrolyte interface. **c** Visual changes in the cell system during discharge in electrolytes containing 0 and 0.3 wt% N-CDs.

To study the optical properties of the as-prepared N-CDs, photoluminescence measurements for the N-CDs in aqueous solution were carried out using different excitation wavelengths (Supplementary Fig. 1). Photoluminescence spectra of N-CDs show the strongest intensity at 380 nm excitation. As shown in Fig. 2a, the N-CDs have excellent dispersibility in the electrolyte of LiTFSI and LiNO_3_ dissolved in the cosolvents of DOL/DME. Even at a high N-CDs content of 5 wt%, no solid precipitation can be found, demonstrating that the N-CDs satisfy the condition of good dispersibility as an ideal electrolyte additive. In contrast, the N-CDs are almost nondispersible/insoluble in the cosolvents of DOL/DME (Supplementary Fig. 2). This might be a result of the strong interaction between the N-CDs and lithium salt (LiTFSI and LiNO_3_) due to the polar oxygen- and nitrogen-containing groups on the surface of the N-CDs (Supplementary Figs. 3, 4 and Supplementary Tables 1, 2), which improves the dispersibility of the N-CDs in the cosolvents of DOL/DME. In this situation, it is desirable to obtain a protective clotting layer via the strong interaction between the N-CDs and LiPS to restrain the subsequent dissolution and diffusion of LiPS. To achieve this goal, Li_2_S_6_ was dissolved in the cosolvents of DOL/DME and added isometrically into the electrolyte containing 0 and 0.3 wt% N-CDs. As shown in Fig. 2b, a LiPS/N-CDs clotting layer is generated immediately upon adding the Li_2_S_6_ solution into the electrolyte with 0.3 wt% N-CDs. More interestingly, there was almost no color or volume change for the electrolyte containing 0.3 wt% N-CDs after 30 min, suggesting that the as-generated clotting layer can effectively prevent Li_2_S_6_ diffusion into the electrolyte. Then, the mixture was violently shaken for 5 min. As shown in Supplementary Fig. 5, it can be seen that the LiPS/N-CDs clotting layer was smashed, and the mixture retained the color of the Li_2_S_6_ solution, demonstrating that not all Li_2_S_6_ participated in the reaction with the N-CDs. In contrast, for the electrolyte without N-CDs, diffusion of the Li_2_S_6_ into the electrolyte is obvious.

To further demonstrate the effect of N-CDs on LiPS clotting and the formation of the LiPS/N-CDs clotting layer, the electrodes (carbon paper) with 2 mg cm^−2^ sulfur loading were discharged at 0.5 mA in the electrolyte containing 0 and 0.3 wt% N-CDs, respectively (Supplementary Fig. 6). During discharge, the electrolyte without N-CDs changed from colorless to bright yellow-green as a result of the dissolved LiPS. By comparison, the 0.3 wt% N-CDs electrolyte remained light brown throughout, suggesting that the generated sulfur species were effectively confined in the cathode.

Therefore, the clotting layer formation could be seen more clearly, and nickel foam with a sulfur loading of 10 mg cm^−2^ was employed as the sulfur cathode and assembled onto the optically transparent Li–S cell. Figure 2c shows visual changes of the electrolyte and sulfur cathode in the optically transparent Li–S cell with 0 and 0.3 wt% N-CDs electrolyte during discharge. For the cell without N-CDs, the color of the electrolyte appears reddish brown after 2 h of discharge, owing to the high concentration of long-chain LiPS. However, for the cell with 0.3 wt% N-CDs electrolyte, the color of the electrolyte after 2 h of discharge is nearly the same as that at the beginning. More importantly, the sulfur cathode is covered with a thin LiPS/N-CDs clotting layer after 1 h of discharge that becomes thicker as the discharge time increases, further confirming that N-CDs possess a significant clotting function for LiPS and that the as-generated LiPS/N-CDs clotting layer suppresses LiPS dissolution and diffusion.

**Fig. 3:**
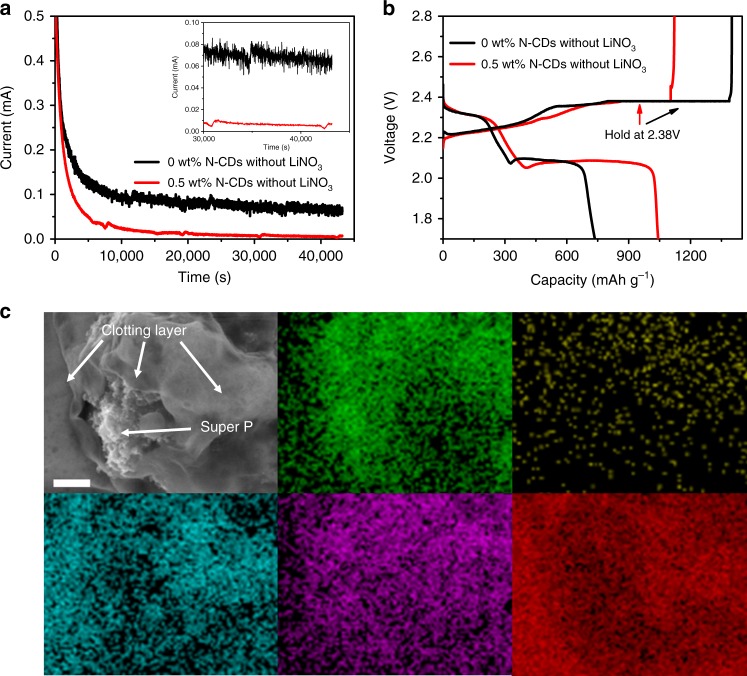
Investigation of the clotting function of N-CDs for LiPS in a 2032 coin cell. **a**, **b** Shuttle current measurement and discharge/charge curves of the coin cells with 0 and 0.5 wt% N-CDs and without LiNO_3_ additive. The shuttle current measurement was held at 2.38 V for 43,200 s in the cycling process at 0.2 C. **c** SEM image and corresponding EDS elemental mapping of the sulfur cathode with 0.5 wt% N-CDs at a discharge voltage of 2.1 V. Green is for C, yellow is for N, blue is for O, purple is for F and red is for S. Scale bar: 1 μm.

Studies have shown that the LiPS concentration is highest at a potential of 2.38 V during the discharge/charge process, resulting in a maximum shuttle current^31,32^. Here, 2032 coin Li–S cells with sulfur loading of ~2 mg cm^−2^ were assembled without adding LiNO_3_ electrolyte, and the shuttle current was measured via 12 h potentiostatic testing at 2.38 V. As shown in Fig. 3a, the shuttle current of the cell with 0.5 wt% N-CDs is almost negligible (7 μA), whereas the cell without N-CDs has a 65 μA shuttle current. It is clear that N-CDs provide considerable clotting function for LiPS, significantly preventing the subsequent dissolution and diffusion of LiPS and leading to enhanced electrochemical performance. As shown in Fig. 3b, the cell without N-CDs delivered low discharge capacity (736 mAh g^−1^) and coulomb efficiency (52%) arising from the fatal shuttling effect of LiPS, whereas the cell with 0.5 wt% N-CDs exhibits superior discharge capacity (1042 mAh g^−1^) and high coulombic efficiency of 93%, which can be ascribed to the clotting effect of N-CDs, significantly blocking the shuttle effect. The cell with 0.5 wt% N-CDs was taken apart when the discharge voltage tapered to 2.1 V, and the surface structure of the sulfur cathode was analysed by scanning electron microscopy (SEM) and energy dispersive spectroscopy (EDS). The LiPS/N-CDs clotting layer covering the super P surface can be clearly observed (Fig. 3c).

### The formation mechanism of the LiPS/N-CDs clotting layer and superior electrochemical performance of Li–S cells with 0.5 wt% N-CDs

As shown in Supplementary Fig. 7a–d, N-CDs with an average diameter of ~5 nm are uniformly dispersed in the electrolyte. The high-resolution transmission electron microscopy image shows that the N-CDs exhibit the characteristic lattice fringes of 0.208 nm, corresponding to the (100) plane of graphite^33,34^. After being mixed with Li_2_S_6_, the N-CDs in the electrolyte agglomerated to form larger clusters of less than 20 nm in size, suggesting that the N-CDs, similar to platelets, can serve as LiPSCFs to form the LiPS/N-CDs clotting layer.

The chemical interaction between the N-CDs and Li_2_S_6_ (as a model compound of LiPS) was studied using X-ray photoelectron spectroscopy. As observed in Supplementary Fig. 7e, the high-resolution S 2p spectrum of Li_2_S_6_ shows two sulfur 2p_3/2_ peaks, at 161.4 and 163.0 eV, with an intensity ratio of 1:2, pointing to the terminal (S_T_^−1^) and bridging sulfur (S_B_^0^) atoms, respectively^23^. After interacting with the N-CDs, the bonding energy of S_T_^−1^ shifts to 161.1 eV, suggesting an increased valence electron density on the sulfur atoms due to the interaction between the N-CDs and Li_2_S_6_^35^. The new peak at 167.2 eV represents the binding energy of thiosulfate, which can be attributed to the surface redox reaction between chemisorbed oxygen on the N-CDs and Li_2_S_6_. Thiosulfate can serve as an internal mediator to anchor and convert long-chain LiPS^36^. Supplementary Fig. 8 presents the FTIR spectra of Li_2_S_6_, N-CDs, and the interaction system of N-CDs and Li_2_S_6_. For pristine Li_2_S_6_, the weak absorption peak at 482 cm^−1^ can be assigned to the typical stretching vibration of the S–S band. The peak position of the S–S band shifted to 503 cm^−1^ in the presence of N-CDs, indicating that the strong interaction between N-CDs and LiPS induces rotation and distortion of the S–S band of absorbed LiPS to form a stable configuration^37^.

**Fig. 4:**
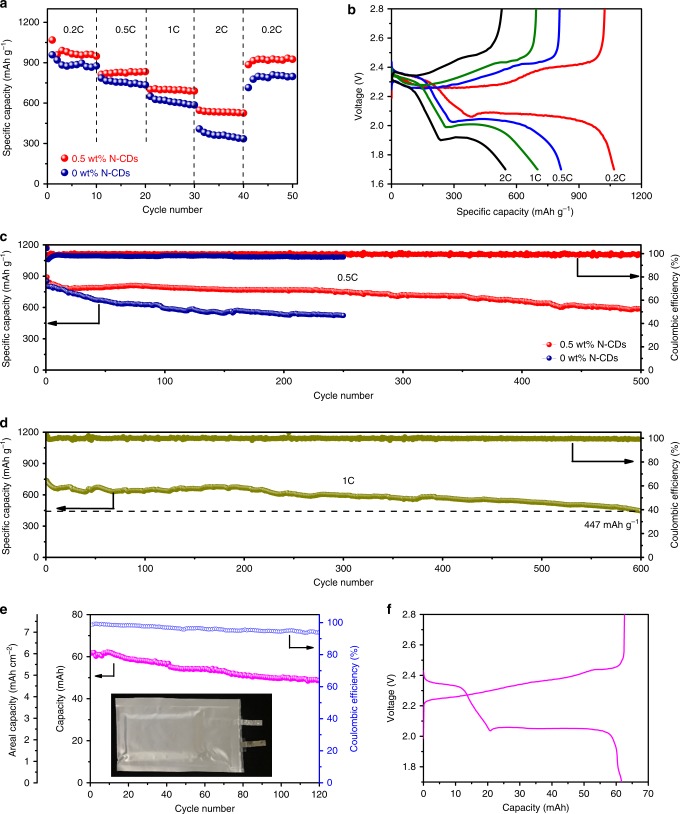
The electrochemical performance of a Li–S cell with 0.5 wt% N-CDs. **a** Rate performance of the cell with 0 and 0.5 wt% N-CDs electrolyte. **b** Voltage profiles of the cell with 0.5 wt% N-CDs electrolyte at different current densities. **c**, **d** Long-term cycling performance of Li–S cells with 0.5 wt% N-CDs electrolyte at 0.5 and 1.0 C. **e**, **f** Galvanostatic cycling performance and voltage profile for the Li–S pouch cell with 0.5 wt% N-CDs electrolyte at 0.2 C.

To evaluate the electrochemical performance of Li–S cells using N-CDs as the electrolyte additive, a series of electrochemical measurements was performed on the assembled 2032 coin cells with sulfur loading of ~2 mg cm^−2^. As shown in Fig. 4a, b and Supplementary Fig. 9, the cell with 0.5 wt% N-CDs electrolyte exhibits higher rate performances than the cells with 0, 0.3, and 1.0 wt% N-CDs electrolyte, especially when the current density is high. The delivered discharge capacity of the cell with 0.5 wt% N-CDs is retained at 1068, 814, and 701 mAh g^−1^ at a current density of 0.2, 0.5, and 1.0 C, respectively. Even at a high current density of 2.0 C, there is still a high reversible capacity of 547 mAh g^−1^. When the current density returns to 0.2 C after 40 cycles, the average discharge capacity is as high as 920 mAh g^−1^, demonstrating the beneficial clotting property of N-CDs for LiPS at high current rates. Supplementary Fig. 10 compares the galvanostatic discharge–charge profiles of Li–S cells with 0 and 0.5 wt% N-CDs electrolyte at 0.2 C. It can be seen that there are no obvious differences in the voltage profile, except for the higher specific capacity for the Li–S cell with 0.5 wt% N-CDs electrolyte. This may be ascribed to the fact that the LiPS/N-CDs clotting layer formed by the interaction between LiPS and N-CDs is just for addressing polysulfide dissolution and diffusion without changing the charge and discharge principles of Li–S batteries. As shown in Supplementary Fig. 11, two typical cathodic peaks at 2.33 and 1.98 V are ascribed to the reduction of sulfur to Li_2_S_*x*_ (4 ≤ *x* ≤ 8) and Li_2_S_2_/Li_2_S, respectively. In the charge process, the overlapping anodic peaks are ascribed to the oxidation of Li_2_S_2_/Li_2_S to Li_2_S_x_ and sulfur. Additionally, the cyclic voltammetry curves almost overlap with each other for the first five cycles, implying superior capacity retention capability and excellent cycling stability.

The long-term cycling performances of the cells with 0 and 0.5 wt% N-CDs were tested at rates of 0.5, 1.0, and 2.0 C, respectively. As shown in Fig. 4c, the cell with 0.5 wt% N-CDs delivers an initial discharge capacity of 891 mAh g^−1^ at 0.5 C. After 500 cycles, a capacity of 589 mAh g^−1^ is retained, corresponding to a low capacity decay rate of 0.067% per cycle with a coulombic efficiency of 99.5%. It is worth noting that at much higher rates of 1.0 and 2.0 C, discharge capacities of 447 mAh g^−1^ over 600 cycles and 348 mAh g^−1^ over 1000 cycles, respectively, are still retained (Fig. 4d and Supplementary Fig. 12). The capacity fluctuations are probably due to the volume fluctuations and redistribution of sulfur during the discharge/charge processes^23^. In contrast, for the cell without N-CDs, the sulfur cathode suffers from much faster capacity decay (0.149% per cycle) even at a low C rate. Moreover, the cell with 6.6 mg cm^−2^ sulfur loading and 0.5 wt% N-CDs still delivers a high areal capacity of 5.8 mAh cm^−2^ and exhibits no obvious capacity decay after 200 cycles (Supplementary Fig. 13).

To further verify the practicability of this strategy, a Li–S pouch cell with 0.5 wt% N-CDs electrolyte was assembled using carbonized cotton fiber foam (CFF)/Li_2_S_6_ with a low specific surface area of 3.1 m^2^ g^−1^ (Supplementary Fig. 14) as the cathode, which can reduce the weight ratio of electrolyte/active sulfur to 8:1^38^. As shown in Fig. 4e, a high area capacity of 6.0 mAh cm^−2^ was achieved. After 120 cycles, the cell can still deliver a quite stable and high areal capacity of 4.7 mAh cm^−2^ due to the N-CDs clotting function for LiPS. Based on the total weight of their components and the average working voltages (Supplementary Table 3), the practical energy density of the Li−S pouch cell was calculated to be 177 Wh kg^−1^.

**Fig. 5:**
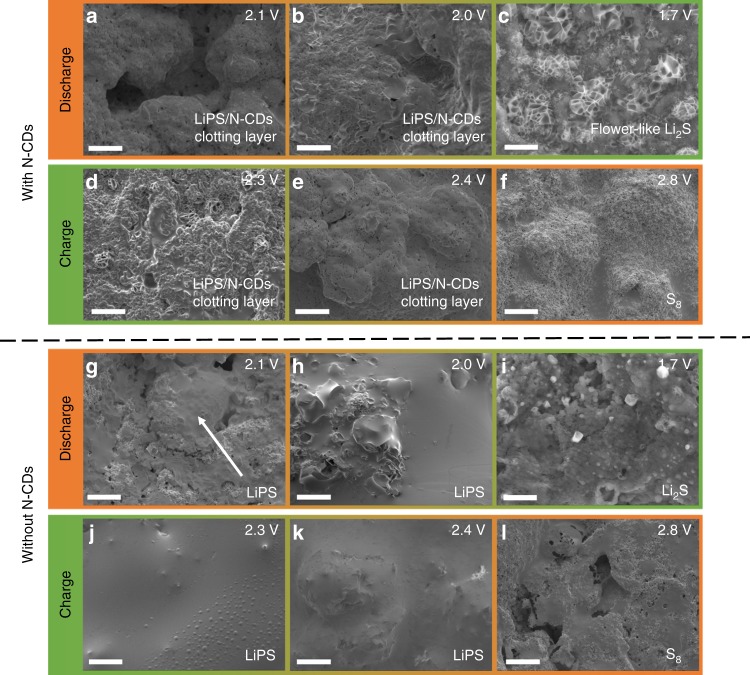
Morphological change of the sulfur cathode surface in coin cells CR2032 loaded with 0 and 0.5 wt% N-CDs electrolyte during the first discharge/charge process. **a**–**f** SEM images of the sulfur cathode with 0.5 wt% N-CDs electrolyte. **g**–**l** SEM images of the sulfur cathode with 0 wt% N-CDs electrolyte. Scale bar: 10 μm.

Morphological changes in the sulfur cathode with 0 and 0.5 wt% N-CDs in the electrolyte during the first discharge/charge process were observed using scanning electron microscopy SEM. At 0.5 wt% N-CDs, the pristine sulfur cathode consists of Super P and sulfur agglomerations before discharge (Supplementary Fig. 15). When the discharge voltage is tapered to 2.1 V, the surface of the sulfur cathode becomes smooth and shiny compared to the pristine sulfur cathode (Fig. 5a), suggesting the formation of a clotting layer of LiPS/N-CDs. Proceeded to discharge to 2.0 V, the surface of the sulfur cathode is mostly covered by a clotting layer of LiPS/N-CDs (Fig. 5b). The elemental mapping images indicate that the clotting layer contains C, N (2.6 wt%), O, F, and S (Supplementary Fig. 16a), implying the coexistence of N-CDs and LiPS in the clotting layer. For the fully discharged sulfur cathode (1.7 V), the LiPS/N-CDs clotting layer is nearly fully replaced by hierarchical porous flower-like Li_2_S crystals (Fig. 5c), which may facilitate rapid electron transfer and ion transport^39^.

A formation mechanism for hierarchical porous flower-like Li_2_S crystals is proposed as follows: the LiPS in the LiPS/N-CDs clotting layer is in situ reduced to Li_2_S, and the N-CDs function as the active sites for Li_2_S crystal growth, giving rise to the temporary disappearance of the clotting layers. This formation mechanism is confirmed by elemental mapping (Supplementary Fig. 16b, c): C and N elements of N-CDs are uniformly dispersed in the flower-like Li_2_S crystals, and the content of N element is as high as 2.2 wt%, indicating that a considerable amount of N-CDs was used as nucleation sites.

During charging, as shown in Fig. 5d, e, the hierarchical porous flower-like Li_2_S crystals are first oxidized to LiPS accompanied by the in situ formation of the LiPS/N-CDs clotting layer via the interaction between LiPS and N-CDs (2.4–2.3 V). For the fully charged sulfur cathode (2.8 V), almost all the LiPS is oxidized to sulfur, causing the disappearance of the LiPS/N-CDs clotting layers and the redispersion of N-CDs into the electrolyte (Fig. 5f). Moreover, there is no noticeable change in the surface morphology of the sulfur cathode even after 50 cycles (Supplementary Fig. 17), suggesting that the introduction of N-CDs can not only restrain the dissolution and diffusion of LiPS but also facilitate the homogeneous electrochemical deposition of insulating sulfur, thus enhancing both the capacity and cycling performance. In contrast, for the sulfur cathode without N-CDs, there is serious dissolution and diffusion of LiPS throughout discharge and charge (Fig. 5g–l), resulting in the loss of active materials from the surface of the sulfur cathode and poor electrochemical performance.

**Fig. 6:**
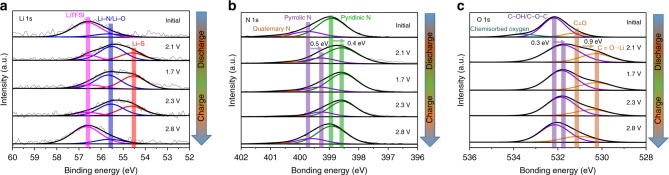
XPS spectra of the sulfur cathode with 0.5 wt% N-CDs electrolyte at different charge/discharge states. **a** Li 1s, **b** N 1s and **c** O 1s.

To further clarify the formation mechanism of the LiPS/N-CDs clotting layer clotting and the existing state of the N-CDs during the different discharge/charge processes for the assembled 2032 coin cells, XPS spectra were obtained for the sulfur cathode of the Li–S cell with 0.5 wt% N-CDs electrolyte at different charge/discharge states. Figure 6a displays the Li 1s high-resolution XPS spectra. For the initial sulfur cathode, the peak at 55.7 eV corresponds to the bonds of Li–N/Li–O^35^, indicative of the strong interaction between LiTFSI and N-CDs. For the sulfur cathode at the state of 2.1 V (discharge), 1.7 V (full discharge) and 2.3 V (charge), there is a new peak located at 54.6 eV, indexed to the Li–S bond, suggesting the presence of LiPS and Li_2_S. Importantly, the intensity of the Li–N/Li–O bond increased significantly compared to that of the initial sulfur cathode, which can be ascribed to the generation of LiPS and Li_2_S, resulting in an increase in the Li–N/Li–O bond. At the intermediate states of discharge (2.1 V) and charge (2.3 V), the as-generated LiPS can interact with the N-CDs to form a LiPS/N-CDs clotting layer at the sulfur electrode–electrolyte interface. At the state of full discharge (1.7 V), the LiPS in the LiPS/N-CDs clotting layer is in situ reduced to Li_2_S, and the N-CDs function as nucleation sites for Li_2_S crystal growth, giving rise to the temporary disappearance of the clotting layers. For the fully charged sulfur cathode (2.8 V), the Li 1s XPS spectra are nearly the same as that of the initial state of the sulfur cathode, implying the renewed dispersion of N-CDs into the electrolyte, which can be further confirmed by the XPS spectra of N 1s and O 1s. As shown in Fig. 6b, c, the peaks of pyridinic N (398.9 eV), pyrrolic N (399.7 eV)^40^, C–OH/C–O–C (532.2 eV) and C=O (531.2 eV)^41^ bonds shift to lower bonding energy at the state of 2.1 V (discharge), 1.7 V (full discharge) and 2.3 V (charge), suggesting the enhancement of the electron cloud density on the N and O atoms owing to the strong interaction between LiPS or Li_2_S and N-CDs. At the fully charged state of 2.8 V, the peaks of N 1s and O 1s shift to the level of the initial state, further confirming the redispersion of N-CDs into the electrolyte.

Previous research has found that pyridinic N exhibits the strongest binding to LiPS among all kinds of doped N species^16,21^. However, the research does not provide a precise model for the simultaneous interaction of LiPS with pyridinic N and oxygen-containing groups simultaneously. It is therefore essential to theoretically understand the interaction of LiPS with both pyridinic N and oxygen-containing groups on the N-CDs using density functional theory.

**Fig. 7:**
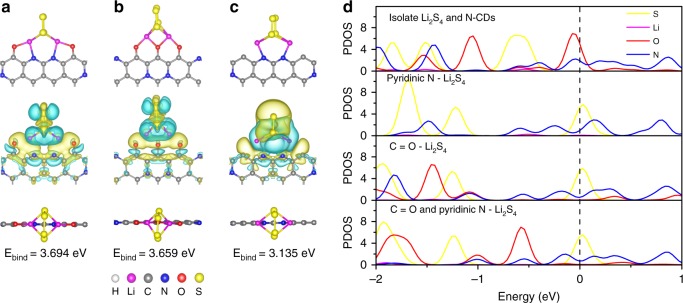
Optimized structures, charge transfers, binding energies, and atomic partial density Li_2_S_4_ absorbed at different sites. **a** C=O and pyridinic N site. **b** C=O site. **c** pyridinic N site. Here, yellow (blue) is the spatial region gain (loss) in charge. **d** Atomic partial density of states near the Fermi energy region for isolate Li_2_S_4_ and N-CDs, Li_2_S_4_ absorbed at pyridinic N and C=O sites, respectively, and Li_2_S_4_ absorbed at C=O sites and pyridinic N sites simultaneously.

Our research found that the configuration of Li_2_S_4_ simultaneously interacting with C=O and pyridinic N is the most favorable structure for the formation of a strongly coupled clotting layer that preserves active materials and restrains the shuttle effect. Figure 7a–c and Supplementary Fig. 18 show the optimized structures and binding energies of Li_2_S_4_ binding to oxygen-containing groups (–C=O, –COOH, C–OH, C–O–C) on the N-CDs and pyridinic N (doped at the center/edge of the N-CDs). Li_2_S_4_ bridging simultaneously to C=O and pyridinic N sites shows the highest binding energy (3.694 eV) of the proposed structures.

This preferred configuration of Li_2_S_4_ simultaneously interacting with C=O and pyridinic N is further confirmed by the differential charge density and the corresponding partial density of states (PDOS). As shown in Fig. 7d, a clear charge transfer from a Li atom to the edge of the N-CDs can be observed, indicating a strong interaction between Li_2_S_4_ and the edge of the N-CDs, which could be mainly caused by the strong electronegativity of oxygen and nitrogen. It can be seen from the PDOS that the O 2p orbitals around the Fermi level are highly unsaturated before the adsorption of Li_2_S_4_. After the adsorption of Li_2_S_4_, the energy levels of the O 2p and N 2p orbitals shift downward, stabilizing the system^42^.

**Fig. 8:**
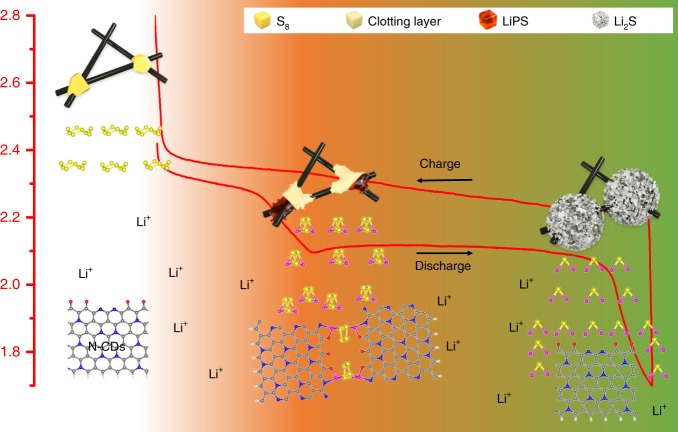
Proposed LiPS clotting mechanism with N-doped carbon dots as the clotting factors of LiPS during the discharge/charge process. The morphology changes of sulfur particles on the surface of carbon fibers as a function of discharge/charge status are depicted in the profile curve. The interaction mechanisms between the N-CDs and LiPS at the interface of the sulfur cathode/electrolyte are portrayed in the lower portion of the figure.

On the basis of our experimental results and theoretical analysis, a possible LiPS clotting mechanism is proposed in Fig. 8. First, the LiPS generated in the process of discharge interact with the N-CDs in the electrolyte to form a LiPS/N-CD clotting layer at the surface of the sulfur cathode, which prevents the subsequent dissolution and diffusion of LiPS and thus solidifies the sulfur cathode. Even though the expansion of the sulfur cathode may crack the initial clotting layer during discharge, causing the diffusion of LiPS, the highly dispersed N-CDs can capture the escaped LiPS and heal the gap. Then, during the discharge process, the LiPS in the LiPS/N-CDs clotting layer are reduced in situ to Li_2_S. With the N-CDs serving as nucleation sites, the as-generated Li_2_S grows into hierarchical porous flower-like Li_2_S crystals, leading to the temporary disappearance of the clotting layers. During the charge process, the evolution of the protective layer is totally reversible. The discharge/charge process is analogous to thrombus formation and thrombolysis in the blood vessel, with N-CDs acting as activated platelets. The LiPS/N-CDs clotting layer can repeatedly generate and dissolve at the surface of the sulfur cathode during discharge/charge, efficiently solidifying the intermediate LiPS and leading to enhanced electrochemical performance.

## Discussion

Inspired by the recoverable processes of thrombus formation and thrombolysis in the blood vessel, we designed N-doped carbon dots as analogous “blood platelets” in Li–S battery electrolytes. Upon the formation of dissolvable LiPS, N-CDs become activated and stimulate the formation of a protective layer at the sulfur electrode–electrolyte interface to suppress sulfur loss. This layer disappears when dissolvable LiPS are absent, as when the electrode is fully charged or discharged. The polysulfide-solidification capability of N-CDs is due to the abundance of N atoms and surface oxygen-containing groups, which strengthen the interaction of N-CDs with LiPS. As a result, high sulfur utilization and long-term cycling stability in Li–S batteries are achieved. We expect the encapsulation concept inspired by blood clotting to also be applied to battery electrochemistry beyond Li–S to prevent the loss of active materials.

## Methods

### Synthesis of N-CDs

The N-CDs were synthesized through rapid one-step pyrolysis of ethanolamine according to the method described in Dong et al.^33^. A mixture of 4.5 mL hydrogen peroxide aqueous solution (30%) and 3.0 mL ethanolamine in a 50 mL beaker was placed into an oven and calcined at 250 °C for 10 min until dark colloidal N-CDs formed.

### Preparation of sulfur cathode

The sulfur cathodes were fabricated using a slurry coating procedure. Sublimed sulfur, Super P, and polyvinylidene fluoride with a mass ratio of 70:20:10 were mixed into N-methyl-2-pyrrolidinone to form homogeneous slurry. The slurry was uniformly coated onto the carbon fibers and dried at 60 °C under vacuum overnight, resulting in an areal sulfur loading of 2 mg cm^−2^. Therefore, the formation of LiPS entrapments could be observed in situ, and a nickel foam electrode with a high areal sulfur loading of ~10 mg cm^−2^ was prepared using the same procedure. In addition, electrodes with a high areal sulfur loading of 6.6 mg cm^−2^ for the coin cell and pouch cell were prepared by adding 0.5 M Li_2_S_6_ in DOL/DME into the carbonized CFF^43^, followed by drying under vacuum overnight. The pouch cell was assembled by using 67.5 mg of S active material and 540 mg of 0.5 wt% N-CDs electrolyte.

### Characterization

Transmission electron microscopy and SEM images of the samples were acquired using an FEI Tecnai G2 F30 STWIN operating at 200 kV and a JEOL 7800F, respectively. X-ray photoelectron spectroscopy analyses were performed using a Thermo Escalab 250 system with a monochromatic Al Kα source (*hν* = 1486.6 eV). Photoluminescent spectra were recorded on a fluorescent spectrophotometer (F-4600, Hitachi). Fourier transform infrared spectra were obtained on a Bruker VERTEC 80v vacuum FT-IR spectrometer. The Bruker–Emmett–Teller (BET) surface area was determined by ASAP 2020 micromeritics.

### Electrochemical measurements

CR2032 coin cells were assembled in an argon-filled glove box (O_2_ and H_2_O levels < 0.1 ppm) for the electrochemical measurements, where the as-prepared sulfur electrode, Celgard 2500 membrane, and Li foil were used as the cathode, separator, and anode, respectively. The electrolyte was 1.0 mol L^−1^ lithium bis (trifluoromethanesulfonyl) imide (LiTFSI) and 1.0% lithium nitrate (LiNO_3_) dissolved in the cosolvents of 1,3-dioxolane (DOL) and 1,2-dimethoxyethane (DME) (v/v = 1:1) with N-CD additives of 0, 0.3, 0.5, 1.0, and 5.0 wt%. The ratio of added electrolyte volume/active sulfur weight was set at 20 μL mg^−1^. Galvanostatic charge–discharge tests were carried out within the cut-off voltages of 1.7−2.8 V using a LAND CT2001A cell test instrument. The current density and the specific capacity were calculated according to the mass of elemental sulfur (1C = 1675 mA g^−1^). For shuttle current measurement, cells with a sulfur loading of ~2 mg cm^−2^ were assembled without adding LiNO_3_. In the cycling process at 0.2 C, the shuttle current of cells was detected via potentiostatic hold on charge at 2.38 V. Cyclic voltammetry measurements were conducted on a Chenhua CHI-760 electrochemical workstation with a scan rate of 0.1 mV s^−1^ in the potential range of 1.7–2.8 V.

### Computational method

Our first-principles calculations were based on density functional theory with generalized gradient approximation for exchange-correlation potential given by Perdew et al.^44^ as implemented in the Vienna ab initio simulation package^45^. The projector-augmented wave method^46^ was used to treat the core electrons, and the valence electrons were represented using the planewave basis set. The planewave cut-off energy was set at 500 eV. The first Brillouin zone was sampled using a Γ-centred Monkhorst-Pack grid^47^. The convergence criteria for energy and force were set to 10^−5^ eV and 0.02 eV/Å, respectively. To simulate the surface adsorption of large molecules, a slab system with a vacuum space of 20 Å along the z direction was adopted. To simulate edge adsorption, a nanoribbon system with a vacuum space of 20 Å along the y and z directions was adopted.

## Supplementary information

Supplementary Information

## Data Availability

All relevant data supporting the key findings of this study are available within the Article, Supplementary Information or available from the authors upon reasonable request.
