# Supplementary material for: Switchable encapsulation of polysulfides in the transition between sulfur and lithium sulfide
Source: Nat Commun. 2020 Feb 12;11:845. doi: 10.1038/s41467-020-14686-2 (PMC7016103; doi:10.1038/s41467-020-14686-2)
Supplement: Supplementary file 1 — Supplementary Information [file 41467_2020_14686_MOESM1_ESM.pdf]

Switchable encapsulation of polysulfide in the transition between sulfur and lithium sulfide

Fu et al.

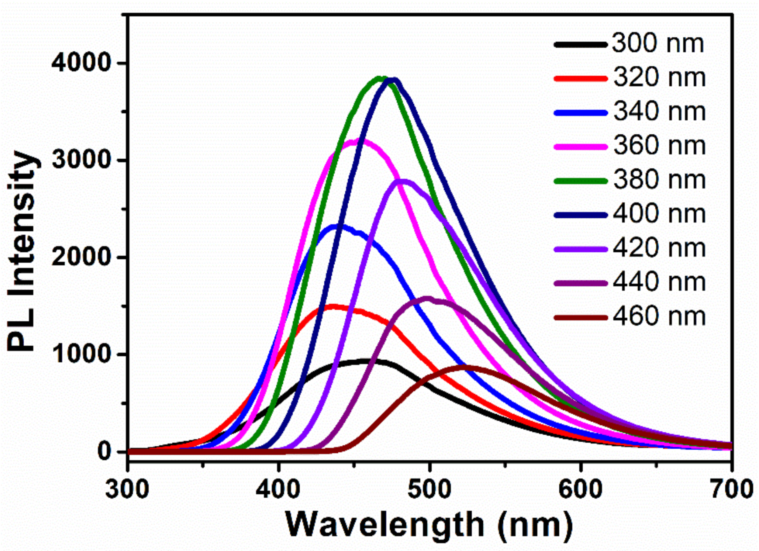

Supplementary Figure 1 | PL emission spectra of N-CDs. PL emission spectra of N-CDs dispersed in water was tested at excitation wavelengths progressively increasing from 300 to 460 nm.

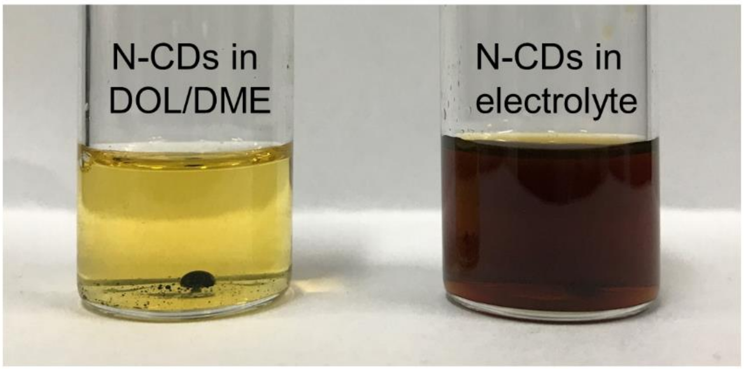

Supplementary Figure 2 | The solubility analysis of N-CDs. Photograph of N-CDs in DOL/DME (left) and electrolyte (right).

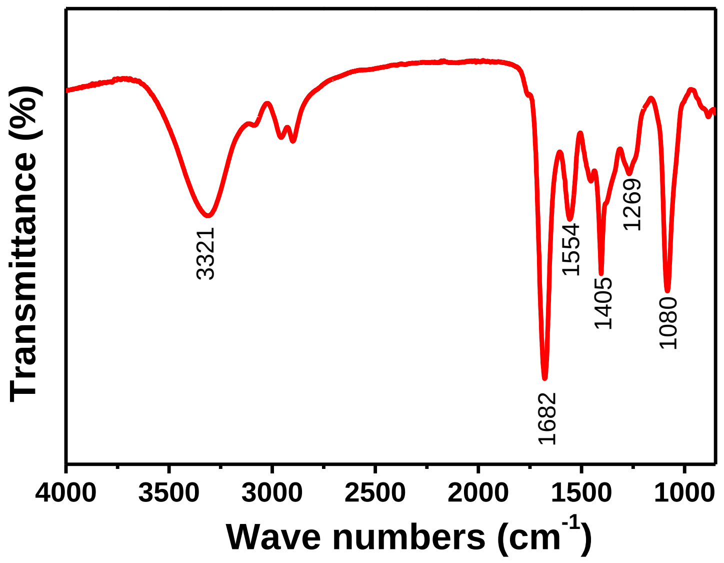

Supplementary Figure 3 | FT-IR spectra of the N-CDs.

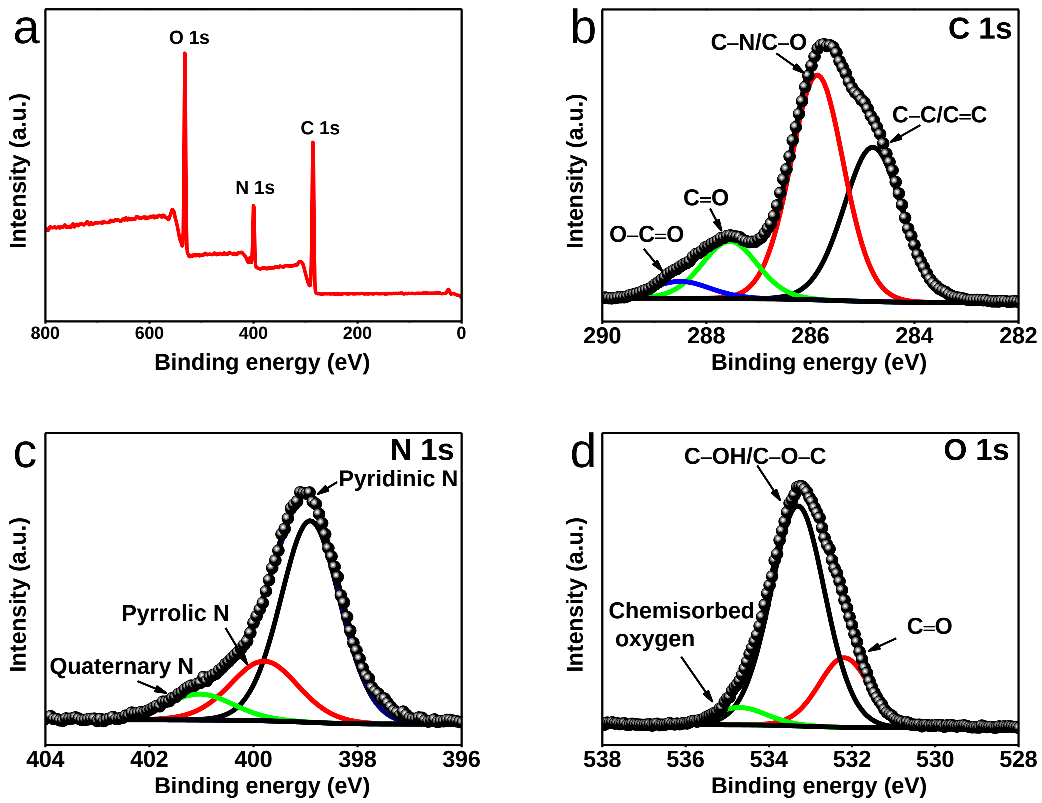

Supplementary Figure 4 | XPS spectra of N-CDs. (a) full-scan, (b) C 1s, (c) N 1s, and (d) O 1s XPS spectra of N-CDs.

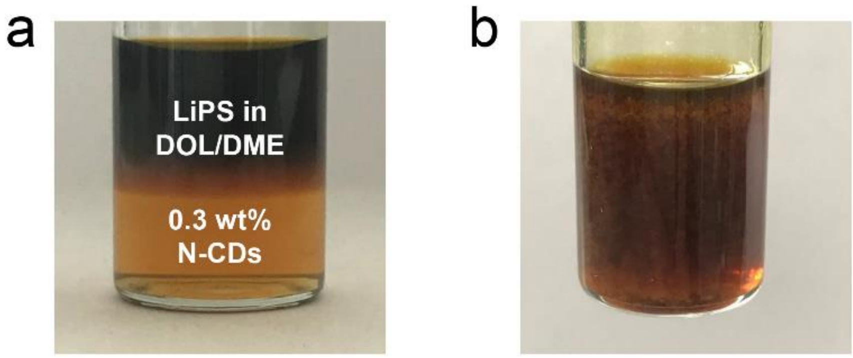

Supplementary Figure 5 | Analysis of the interactions between LiPS and N-CDs. Photographs of (a) LiPS/N-CDs clotting layer formation and (b) the well mixed mixture.

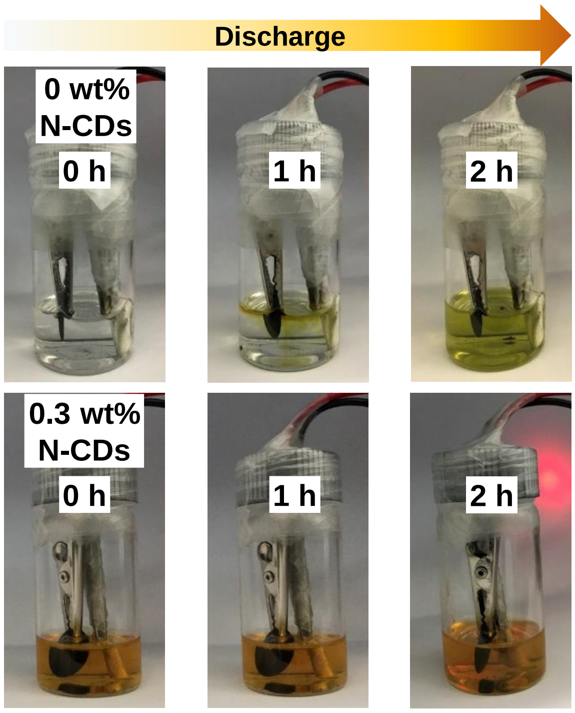

Supplementary Figure 6 | Visual cell tests for confirming polysulfide entrapment. (a) Commercial electrolyte and (b) 0.3 wt% N-CDs electrolyte cells were both discharged at 0.5 mA using carbon fiber electrodes.

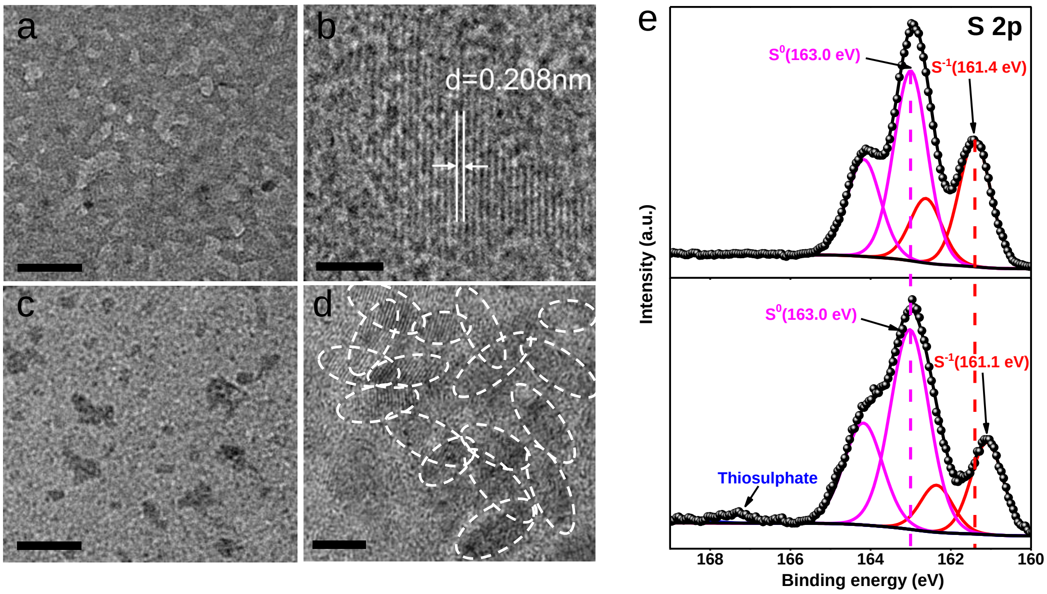

Supplementary Figure 7 | TEM and XPS analysis. (a and b) TEM and HRTEM images of N-CDs, (c and d) low- and high-magnification TEM images of N-CDs mixed with Li_2S_6, and (e) S 2p core-level spectra of Li_2S_6 before and after interacting with N-CDs. Scale bar: 20 nm (a), 2 nm (b), 20 μm (c), 5 nm (d).

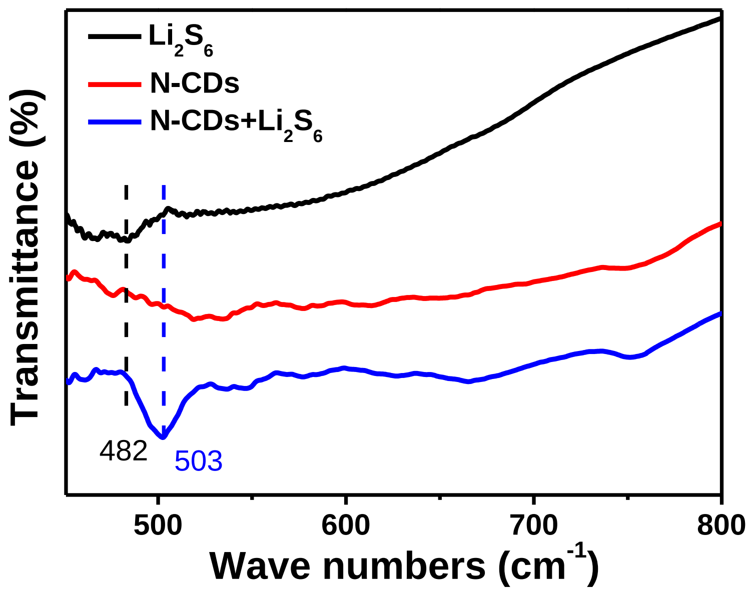

Supplementary Figure 8 | FTIR analysis. FTIR spectra of Li_2S_6, N-CDs, and N-CDs + Li_2S_6.

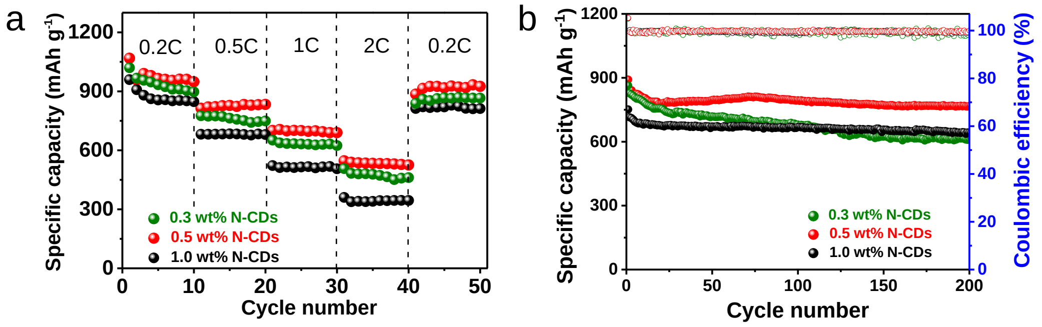

Supplementary Figure 9 | The electrochemical performance of the cells with different N-CDs contents. (a) Rate performance and (b) long-term cycling performance of the Li–S cells with 0.3, 0.5 and 1.0 wt% N-CDs electrolyte at 0.5 C.

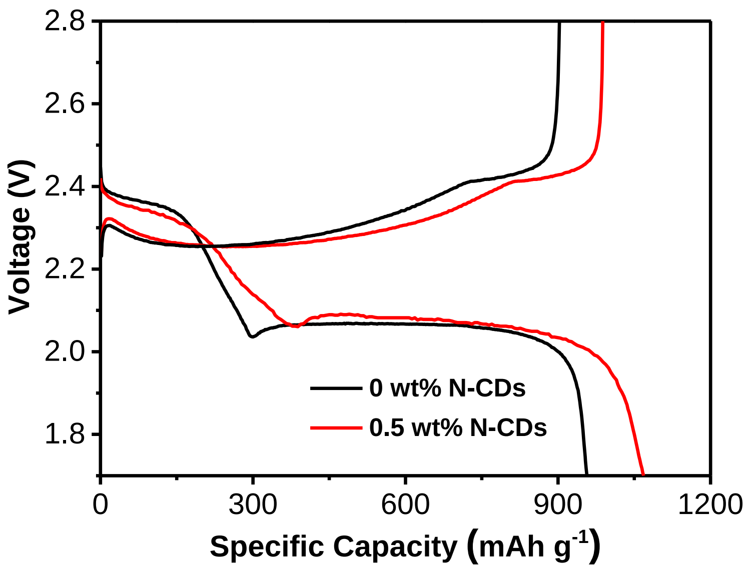

Supplementary Figure 10 | Voltage profile of the cells. Galvanostatic discharge–charge profiles of the Li–S cells with 0 and 0.5 wt% N-CDs electrolyte at 0.2 C.

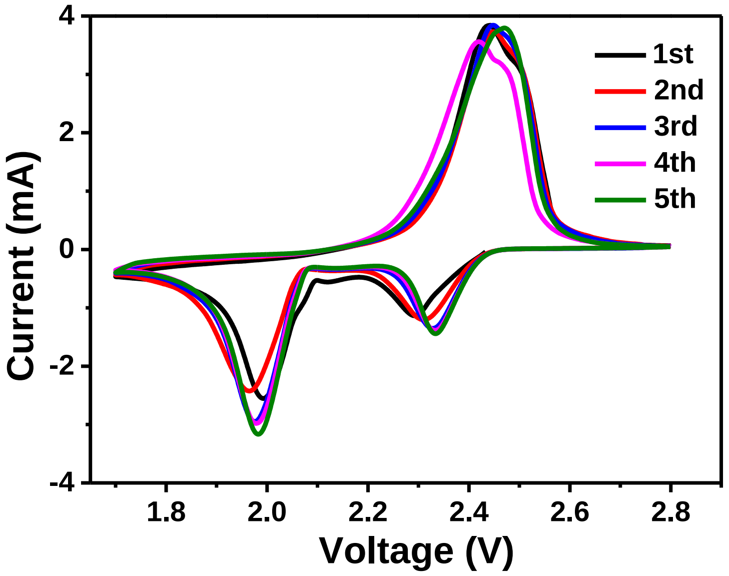

Supplementary Figure 11 | CV profiles of the cell with 0.5 wt% N-CDs electrolyte. CV measurement was conducted in the voltage range of 1.7–2.8 V at a sweep rate of 0.1 mV s^{-1}.

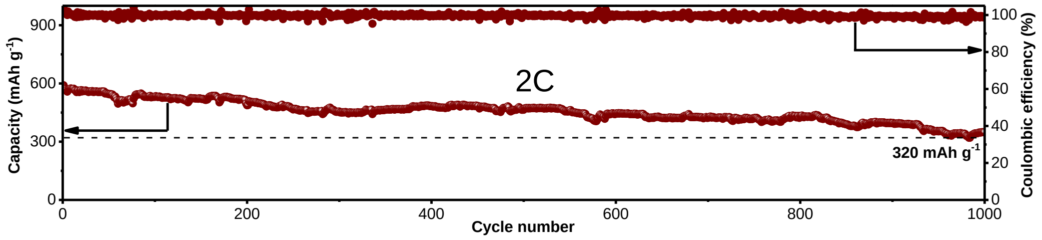

Supplementary Figure 12 | Electrochemical measurement. Long-term cycle performances of Li-S cells with 0.5 wt% N-CDs electrolytes at 2 C.

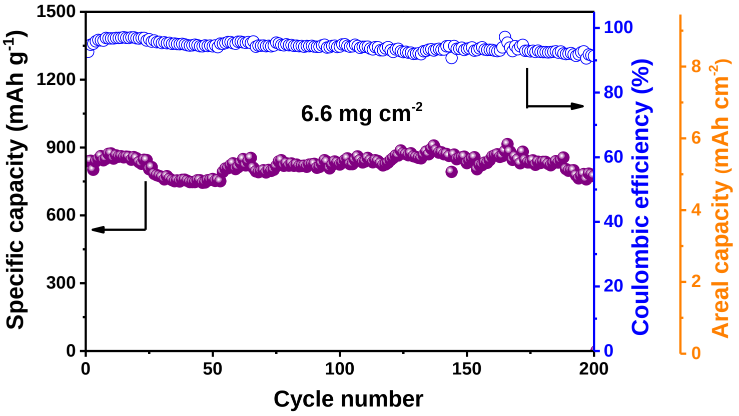

Supplementary Figure 13 | Electrochemical measurement. Cycling performance of high-sulfur-areal-loading cell with 0.5 wt% N-CDs electrolyte at 0.2 C.

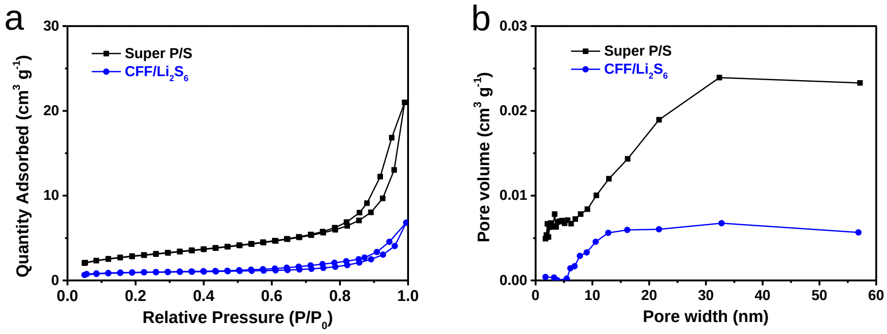

Supplementary Figure 14 | N_2 adsorption/desorption analysis. (a) N_2 adsorption-desorption isotherms, and (b) corresponding pore-size distributions of the Super P/S and CFF/Li_2S_6 electrodes.

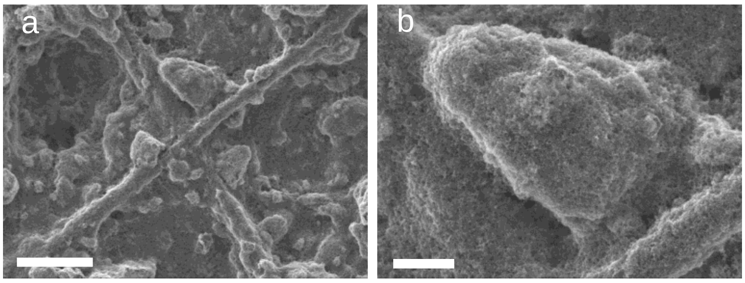

Supplementary Figure 15 | SEM image of the pristine sulfur cathode. Scale bar: 50 µm (a), 10 µm (b).

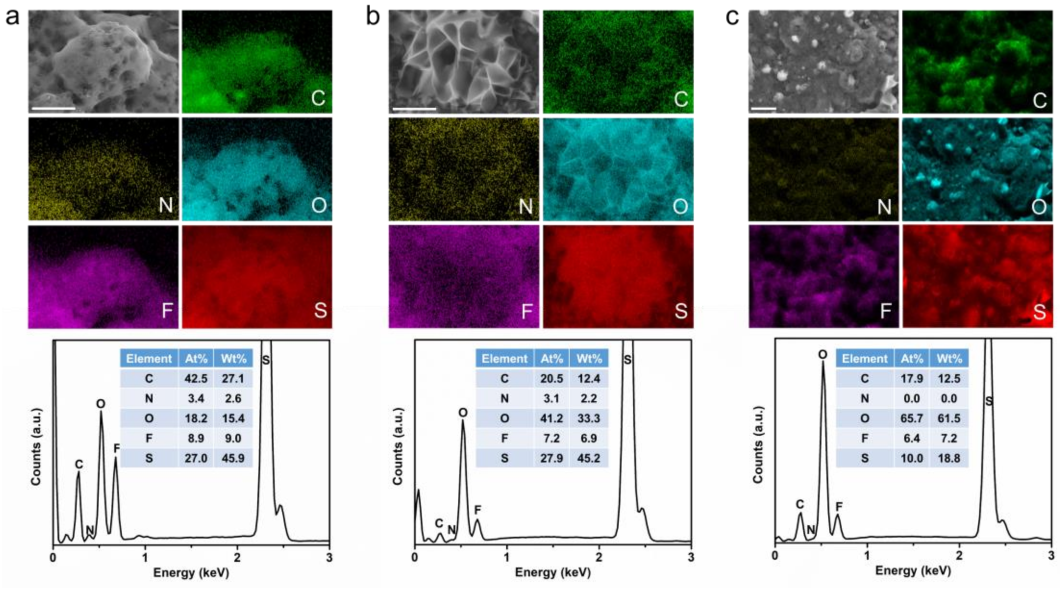

Supplementary Figure 16 | Morphology characterizations of the cells at discharged state. SEM image and corresponding EDS elemental mapping of (a) sulfur cathode using 0.5 wt% N-CDs electrolyte when the discharge voltage is raised to 2.0 V, (b) fully discharged sulfur cathode with 0.5 wt% N-CDs electrolyte, and (c) fully discharged sulfur cathode with 0 wt% N-CDs electrolyte. Scale bar: 5 μm (a), 5μm (b), 10 μm (c).

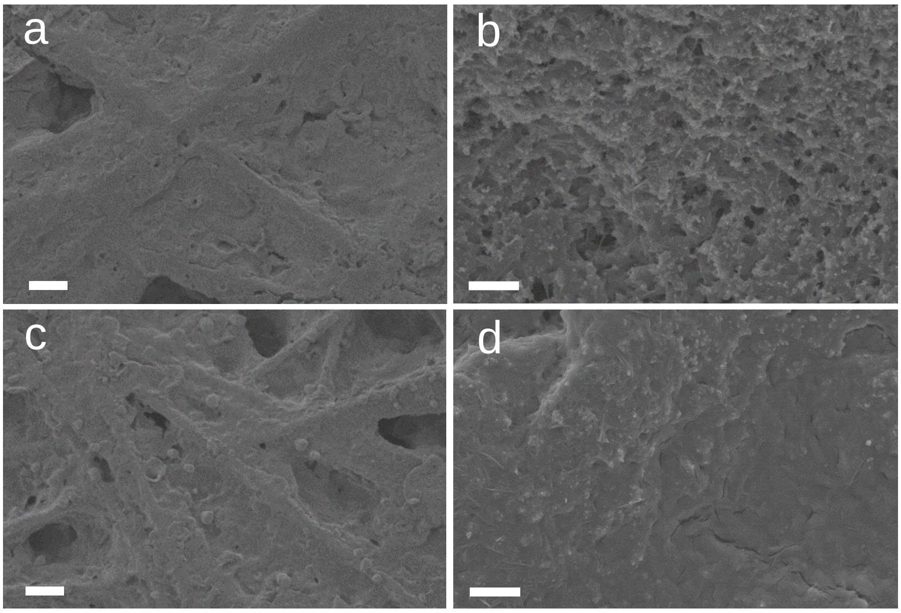

Supplementary Figure 17 | Morphology characterizations of the cells after 50 cycles. SEM images of (a and b) the sulfur cathode without N-CDs after 50 cycles, and (c and d) the sulfur cathode with 0.5 wt% N-CDs electrolyte after 50 cycles. Scale bar: 50 μm (a), 2 μm (b), 50 μm (c), 2 μm (d).

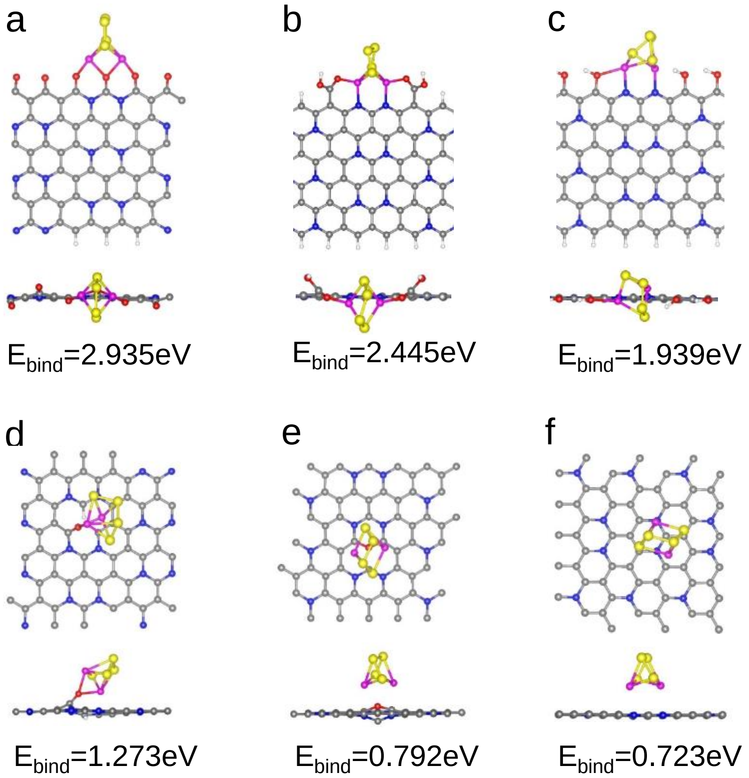

Supplementary Figure 18 | The optimized structures and binding energies of Li_2S_4 at various active sites. (a) Li_2S_4 at the C=O site, (b) Li_2S_4 at the –COOH site, (c and d) Li_2S_4 at the –OH site, (e) Li_2S_4 at the C–O–C site, and (f) Li_2S_4 at the pyridinic N site.

Supplementary Table 1 | Elemental content (at.%) of N-CDs according to XPS analysis.

Elements	C	O	N
Content	57.7	27.4	14.9

Supplementary Table 2丨N species content (at.%) of N-CDs based on XPS analysis.

Groups	Pyridinic N	Pyrrolic N	Quaternary N
Content	67.1	22.8	10.1

Supplementary Table 3 | Values used for the calculation of the practical gravimetrical energy density of the assembled Li-S cell.

V	C	m_A	$W_{Celgard}$	$W_{lithium}$	$W_{electrolyte}$	W_{CFF}
2V	913 mAh g^{-1}	6.6 mg cm^{-2}	1.26 mg cm^{-2}	4 mg cm^{-2}	52.8 mg cm^{-2}	3.5 mg cm^{-2}

Supplementary Notes

As shown in Supplementary Fig. 3, the FT-IR peaks for N-CDs may be assigned as O–H stretching vibrations of C–OH (3,321 cm^{-1}); C=O stretching vibrations of COOH linked to the benzene ring or $(C)_2$–C=O in the benzene ring (1,682 cm^{-1}); C–O stretching vibrations of the epoxy groups (1,080 cm^{-1}); C–N/C=N stretching modes heterocycles (1,405 and 1,269 cm^{-1}); and C=C stretching vibrations in the benzene ring (1,554 cm^{-1}).[1] These FT-IR features indicate that the N element is successfully doped onto carbon dots while the oxygen-containing groups also exist on the dots.

The XPS survey spectrum shows that N-CDs contain C, N, and O as the primary elements (Supplementary Fig. 4a). The C, O, and N contents were calculated from the XPS results and are listed in Supplementary Table 1. It can be seen that the N content is as high as 14.9% as a result of the longer pyrolyzing reaction time for ethanolamine. As shown in Supplementary Fig. 4b, the C 1s spectrum can be deconvoluted into four C species: the peaks at 284.8, 285.9, 287.6, and 288.5 eV, which can be assigned to C–C/C=C, C–N/C–O, C=O, and O–C=O, respectively.[2,3] In addition, the high-resolution N 1s spectrum was deconvoluted into three peaks corresponding to pyridinic N(398.9 eV), pyrrolic N(399.8 eV), and quaternary N (401.0 eV), respectively (Supplementary Fig. 4c).[4] From Supplementary Table 2, it can be seen that the pyridinic N is predominant in all of the N-species. Supplementary Fig. 4d shows the high-resolution XPS spectrum of O 1s, which can be fitted into three components: the main peak at 533.3 eV is ascribed to C–OH/C–O–C, while another two weak peaks at 532.4 and 534.7 eV can be attributed to C=O and chemisorbed oxygen, respectively.[5] The high content of pyridinic-N and C=O groups may promote the interaction between N-CDs and LiPS, thus preventing the subsequent dissolution and diffusion of LiPS.

In the new Li-S pouch cell with 0.5 wt% N-CDs electrolyte, the weight ratio of electrolyte/active sulfur can be reduced to 8:1 using carbonized cotton fiber foam (CFF)/Li_2S_6 as sulfur cathode. The practical energy density of Li-S pouch cell was calculated to be 177 Wh kg^{-1} according to the following equation:[6]

$$Eg = \frac{V \cdot m_{\mathrm{A}} \cdot C}{\sum W_{\mathrm{i}}}$$

where: Eg (Wh kg^{-1}) is the specific energy density, V (V) is the average cell operating voltage, m_A (g cm^{-2}) is the active material loading, C (mAh g^{-1}) is the active material capacity, W_i is the weight of individual cell components (g cm^{-2}).

For the sulfur cathode of coin cell CR2032 without N-CDs, the active sulfur materials can transfer to the surface of the electrode and exhibit a rough and porous structure after 50 cycles (Supplementary Fig. 17a and b), which can be ascribed to the severe dissolution and diffusion of LiPS. By contrast, for the sulfur cathode of coin cell CR2032 with 0.5 wt% N-CDs electrolyte after 50 cycles (Supplementary Fig. 17c and d), the skeleton of carbon fiber current collector can be clearly observed, similar to that of the pristine sulfur cathode before charge/discharge. Active sulfur materials are distributed uniformly on the surface of the current collector, indicating that the introduction of N-CDs facilitates the homogeneous electrochemical deposition of insulating sulfur.

Supplementary References

1. Dong, X. et al. Fast one-step synthesis of N-doped carbon dots by pyrolyzing ethanolamine. *J. Mater. Chem. C* **2,** 7477−7481 (2014).

2. Yang, Z. et al. Nitrogen-doped, carbon-rich, highly photoluminescent carbon dots from ammonium citrate. *Nanoscale* **6,** 1890−1895 (2014).

3. Yuan S. et al. Graphene-supported nitrogen and boron rich carbon layer for improved performance of lithium–sulfur batteries due to enhanced chemisorption of lithium polysulfides. *Adv. Energy Mater.* **6,** 1501733 (2016).

4. Li F. et al. The synergistic effect of pyridinic nitrogen and graphitic nitrogen of nitrogen-doped graphene quantum dots for enhanced TiO_2 nanocomposites' photocatalytic performance. *Catalysts* **8,** 438 (2018).

5. Oh, Y. J. et al. Oxygen functional groups and electrochemical capacitive behavior of incompletely reduced graphene oxides as a thin-film electrode of supercapacitor. *Electrochim. Acta* **116,** 118−128 (2014).

6. Agostini, M., et al. Minimizing the electrolyte volume in Li–S batteries: a step forward to high gravimetric energy density. *Adv. Energy Mater.* **8,** 1801560 (2018).
